# Knowledge and Beliefs Toward Mammography Screening Among Jordanian Women: Cross-Sectional Study

**DOI:** 10.2196/75384

**Published:** 2025-08-21

**Authors:** Ahmad Shaker Abuabed, Hana Taha, Luz María Garcia-Valdes, Mohammad Al-Share, Hadi Al-shar'e, Carmen Amezcua-Prieto

**Affiliations:** 1 Department of Preventive Medicine and Public Health, Faculty of Medicine, University of Granada, Granada, Spain; 2 Department of Family and Community Medicine, School of Medicine, University of Jordan, Amman, Jordan; 3 Department of Neurobiology, Care Sciences and Society, Karolinska Institutet, Nobels väg 6, Stockholm, 171 77, Sweden, 46 852480000; 4 General Surgery Department, General Surgery Department, Al-Bashir Hospital, Amman, Jordan; 5 General Practitioner, Private Clinic, Amman, Jordan; 6 Madrid, CIBER of Epidemiology and Public Health, Carlos III Health Institute, Granada, Spain; 7 Instituto de Investigación Biosanitaria de Granada (IBS.GRANADA), Granada, Spain

**Keywords:** breast cancer, mammogram screening, beliefs, champion’s health belief model, knowledge

## Abstract

**Background:**

Breast cancer (BC) is the most commonly diagnosed cancer and the leading cause of cancer-related deaths among women globally. Despite the significance of mammography screening rate for early BC detection among Jordanian women, it remains low, mainly due to various cognitive, psychosocial, and behavioral barriers. Understanding these factors is essential for developing effective interventions.

**Objective:**

This study aims to assess the BC knowledge and beliefs about mammography screening among Jordanian women aged 40 years and older based on the Health Belief Model (HBM) as a theoretical framework.

**Methods:**

A cross-sectional design was used with a convenience sample of eligible women from a Jordanian public hospital. Data were collected through face-to-face interviews using a validated Arabic structured questionnaire consisting of 3 sections: sociodemographic data, knowledge about BC, and health beliefs about mammography. Descriptive statistics and multivariate analysis of variance (MANOVA) were conducted using IBM SPSS version 28.

**Results:**

A total of 405 women completed the study, with an average (SD) age of 52.4 (8.57) years. Findings revealed a notably low knowledge level, as participants scored an average (SD) of 5.80 (2.64) out of 12. The average (SD) scores for the health beliefs section (out of 5) were also low: perceived benefits, 2.59 (0.59); perceived barriers, 2.48 (0.71); and health motivation, 2.51 (0.71). Significant associations (*P*<.001) with medium to large effect sizes (ηp²>0.06) were observed between participants’ age and education level in relation to BC knowledge and health beliefs regarding mammography screening. Participants cited several reasons for their reluctance to undergo mammography, including a lack of knowledge (72.8%), cultural beliefs (63%), and religious factors (29.4%).

**Conclusions:**

A significant gap exists in BC knowledge and beliefs about mammography among Jordanian women aged 40 years and older. Policy makers and health care providers should prioritize the development of tailored strategies and context-specific, sensitive educational interventions. These efforts should address the unique needs, cultural beliefs, and awareness levels of this population to improve mammogram screening practices in Jordan.

## Introduction

### Background

Cancer has become a significant global public health concern, surpassing cardiovascular diseases as the leading cause of death in many high-income countries [1]. Breast cancer (BC) is the most diagnosed, with around 2.3 million new cases and 670,000 deaths in 2022 [2-4], making it the leading cause of cancer-related death among women globally [3,4]. Although the incidence of BC is lower in high-income countries, it is often diagnosed at advanced stages, leading to higher mortality rates [5].

As a low-income country, the World Bank classifies Jordan as a lower-middle-income country (LMIC) [6]. It confronts substantial health care resource management challenges, particularly for noncommunicable diseases like cancer [5,7]. BC is the most common cancer among women in Jordan, accounting for 39.2% of female cancer cases in 2022. The age-standardized rate (ASR) of BC is 60 per 100,000 population, which is higher than in some neighboring Arab countries but lower than in Western countries. The global ASR average is 46.8 [2,8,9]. The rising incidence of BC in LMICs is linked to increased life expectancy, improved health care services, and increased health insurance coverage. In addition, the adoption of Western lifestyles and changes in reproductive behaviors, such as delayed childbirth and shorter breastfeeding durations, have also contributed to this trend [10,11].

The American College of Radiology (ACR) recommends that all women at average risk of BC should begin annual mammograms at the age of 40 years. Moreover, women who are at higher-than-average risk due to various factors are recommended to undergo more intensive screening [12]. Ultimately, mammography screening can reduce BC mortality by 40% or more in women aged 40 years and older [12]. Early detection of BC through mammography can lead to significantly improved survival rates [12,13]. In response to this, Jordan established the Jordan Cancer Registry in 1996, the King Hussein Cancer Foundation and Center in 2001, and the Jordan Breast Cancer Program (JBCP) in 2007 to coordinate detection efforts, provide screening, and raise public awareness [11]. It also recommends that women begin mammogram screenings at the age of 40 years [9,14].

Despite these national initiatives, participation in mammography screening remains low. According to the Jordan Cancer Registry, only 18.6% of women aged 40-69 years have undergone mammographic screening in the past 2 years [15]. Another survey indicated that just 14% of women aged 40-49 years have ever had a mammogram [16]. These rates are comparable to those in neighboring countries, such as Qatar (22.5%) and Saudi Arabia (27.7%) [17,18]. However, they are still significantly lower than the rates reported in high-income countries, such as France (48.5%) and the United States (61%) [19,20]. This low participation persists despite the expansion of screening services and public awareness campaigns, indicating ongoing barriers to preventive care [7,11,14].

One of the main challenges and barriers is that many Jordanian women are unaware of their eligibility for screening due to a lack of awareness, absence of health problems, fear of diagnosis, and the influence of cultural and religious beliefs [16,21]. Although educational interventions have been implemented, evidence suggests that simply increasing awareness is insufficient to effect meaningful change in screening behaviors [21-23]. Deeply rooted beliefs and attitudes, shaped by cultural and individual factors, continue to hinder adherence to breast cancer screening (BCS) guidelines [24,25]. Furthermore, health beliefs regarding mammography among this age group in Jordan are underresearched [26,27].

To address these gaps, this study aims to investigate how knowledge and health beliefs about mammography screening practices based on the Health Belief Model (HBM) among Jordanian women aged 40 years and older. The study also seeks to identify the sociodemographic characteristics that influence knowledge and beliefs regarding screening.

### Theoretical Framework

This study is guided by the HBM, a theoretical framework that explains preventive health behaviors in various contexts, such as vaccination and smoking [28,29]. The HBM suggests that individuals’ actions are influenced by their perceptions of susceptibility, severity, benefits, barriers, cues for action, and self-efficacy [28-30].

Victoria Champion developed the Champion Health Belief Model (CHBM) as an adaptation of the original HBM to better understand and predict women’s participation in BCS behaviors [31,32]. Champion initially created separate scales to measure constructs such as perceived susceptibility, benefits, and barriers related to BCS [31,32]. These scales were later combined and revised to form the Champion Health Belief Model Scale (CHBMS) [33,34]. This comprehensive instrument emphasizes health motivation and self-efficacy in women’s decisions to participate in BCS, while also taking into account cultural factors [33,34]. The CHBMS has been adapted and validated in various cultural contexts to assess the beliefs and attitudes that influence BCS behaviors [22,26,34,35].

In this study, we used 3 constructs of the CHBMS: perceived benefits, barriers, and health motivation. These constructs were selected based on the study conducted by Petro-Nustas [26], who revised these specific aspects of the CHBMS [22,33]. This psychometrically validated tool has been used to assess the beliefs of Jordanian women regarding mammography.

## Methods

### Study Design

This study implemented a cross-sectional design to assess the knowledge and beliefs about BC and mammography among Jordanian women aged 40 years and older. It adhered to the Strengthening of the Reporting of Observational Studies in Epidemiology (STROBE) guidelines (Checklist 1) [36]. The HBM served as the theoretical framework for this study, as it has been identified as the most frequently used and suitable theoretical health behavior model in previous studies that explain women’s BCS practices [37,38].

### Study Setting

This study was conducted at Albashir Hospital in Amman, Jordan, the largest public medical complex in the country. It is a national referral center comprising multiple specialized hospitals and centers. The sample included women from different socioeconomic and clinical backgrounds. In 2022, the hospital’s outpatient clinics received a total of 632,352 patients who visited the hospital and the outpatient clinics. Participant recruitment and data collection took place from women attending various outpatient clinics between May and July 2024 during regular clinic hours (8:00 AM to 4:00 PM, Sunday through Thursday).

### Participants

#### Eligibility Criteria

Women of Jordanian nationality who are 40 years of age and older were eligible to participate in this study, provided they met specific criteria. Participants were required to demonstrate sufficient literacy in Arabic, have not received mammography screening within the previous 12 months, and be free from any physical or mental disabilities that could impede independent participation or reliable self-reporting. To maintain the study’s focus on screening behaviors, individuals with a personal history of BC or those who have a first-degree relative (parent, sibling, or child) diagnosed with BC were excluded from participation.

#### Selection and Recruitment

A convenience sampling strategy was used, with a multiphase approach to uphold ethical standards, ensure participant privacy, and maintain methodological rigor. Before the recruitment process began, all health care providers involved in the recruitment and data collection team participated in a 3-day training in May 2024. This training addressed the study’s methodology, objectives, questionnaire elements, and ethical considerations to ensure consistent data collection and minimize interviewer bias.

Each morning, the appointment schedules at the various outpatient clinics were reviewed to identify women eligible for the study, thereby ensuring a diverse population and minimizing selection bias. Team members approached these women in the waiting areas, explaining the research and inviting them to participate while ensuring that they minimized any disruption to their time. Interested women were then escorted to a private meeting room, where they received detailed information and reassurances that their participation would not impact their health care.

Women had the right to decline or provide written consent if they agreed to participate. After obtaining consent, face-to-face interviews were conducted by the same team member who initiated recruitment, ensuring consistency and rapport with the participants. To support women with low literacy levels, each interview followed a structured questionnaire format. The interviewer offered additional clarification and read the questions aloud as needed, ensuring that all participants, regardless of their literacy level, could fully understand and answer each item. The interviews, conducted in Arabic, typically lasted between 10 and 15 minutes to reduce information bias.

### Data Sources and Measurement

The variables of interest in this study—sociodemographic data, knowledge of BC, and health beliefs about mammography screening—were clearly defined and systematically measured. Data for all variables were collected using a questionnaire. The questionnaire consisted of 3 sections: sociodemographic characteristics, knowledge about BC, and health beliefs, measured using 3 constructs from the CHBMS.

The sociodemographic data section included questions about participants’ age, education, marital status, health insurance status, and mammography screening history in the past 12 months (these questions were used to assess participants’ eligibility). In addition, participants were asked an open-ended question to provide up to 4 reasons for not having a mammogram, offering qualitative insight into the barriers to screening, with no specific order required.

The knowledge section featured 12 true, false, or do not know questions about BC, with 7 correct answers marked as “true” and 5 as “false.” Participants scored 1 point for each correct answer and zero for each incorrect or “do not know” answer. The maximum possible score was 12, indicating excellent knowledge, while a score of zero indicated limited knowledge.

The health belief section consisted of 3 constructs from the CHBMS, comprising 18 statements: 6 related to perceived benefits, 5 to perceived barriers, and 7 to health motivation. The scale is positively associated with screening behaviors, except for perceived barriers, which are negatively associated. Each statement was rated on a 5-point Likert scale (1=strongly disagree to 5=strongly agree), with reversed scoring applied to the barriers. Scores ranged from 18 (highly negative beliefs) to 90 (highly positive beliefs).

In the previous section on health beliefs, we used a culturally adapted Arabic version of the CHBMS (Multimedia Appendix 1), initially translated and validated for BCS among Jordanian women by Mikhail and Petro-Nustas in 2001 [35]. Petro-Nustas later revised and validated the entire questionnaire to better reflect the health beliefs of Jordanian women regarding mammography, focusing on 3 constructs from the CHBMS: perceived benefits, perceived barriers, and health motivation [22]. Our study followed this methodology and obtained permission from Petro-Nustas and Champion to use the questionnaire (Multimedia Appendix 2).

### Study Size

The study size was determined based on previous relevant studies [14,24,27] 27,27 and calculated using the Raosoft sample size calculator (Raosoft, Inc) [39]. The target population consisted of Jordanian women aged 40 years or older, totaling approximately 1,460,300 in 2023. Based on a 95% confidence level, a 5% margin of error, and a response distribution of 50%, the minimum required sample size was determined to be 384 participants. A total of 405 women were recruited to mitigate potential nonresponses, attrition, and data exclusion while preserving statistical power.

### Statistical Analysis

Descriptive statistics were calculated, including the mean and SD for continuous data and frequencies and percentages for categorical data. A Multivariate Analysis of Variance (MANOVA) was conducted to examine the association between sociodemographic independent variables: age, educational level, marital status, and health insurance, and the dependent variables: knowledge and health beliefs based on the HBM, specifically in terms of perceived benefits, perceived barriers, and health motivation. Following significant results from the MANOVA (*P*<.05), indicating an association between the dependent and independent variables, Bonferroni post hoc tests were conducted to determine which specific groups within each dependent variable showed statistically significant differences (*P*<.001); to quantify the magnitude of effects beyond statistical significance, partial eta-squared (ηp²) values were calculated for each independent and dependent variable relationship. All statistical analyses were performed using IBM SPSS version 28 [40].

### Ethical Considerations

This study was conducted in accordance with the ethical principles outlined in the Declaration of Helsinki. Ethical approval was obtained from the Institutional Review Board (IRB) of the Ministry of Health and Albashir Hospital in Jordan (IRB-7790) in May 2024 (Multimedia Appendix 3). Written consent was obtained from all participants before their participation (Multimedia Appendix 4). They were provided with detailed information about the study’s aims and significance, as well as their right to withdraw at any time without any obligations. To ensure privacy, no personal identifiers were used; participants were assigned unique code numbers to maintain confidentiality. No financial compensation or incentives were provided to participants.

## Results

### Overview

**Figure 1.**
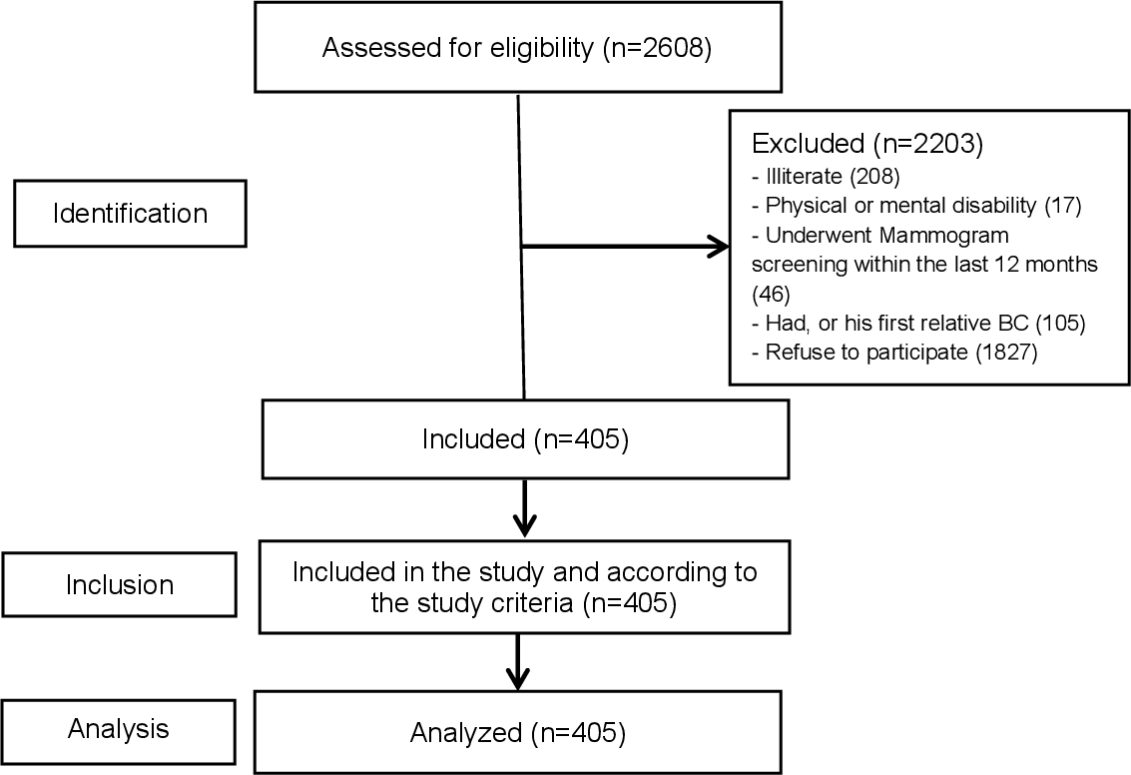
Strengthening the Reporting of Observational studies in Epidemiology flow diagram

Out of 2608 women screened at Albashir Hospital outpatient clinics, 376 were excluded, and 1827 refused to participate, resulting in 405 eligible participants who consented and completed the study. This process is outlined in the STROBE flow diagram [36] as shown in Figure 1.

### Sociodemographic Characteristics

A total of 405 Jordanian women participated in the study, aged 40-76 years. The vast majority, 187 (46.2%), fell within the 40‐49 years age group, 173 (42.7%) completed secondary education, 213 (52.6%) were married, 372 (91.9%) reported having health insurance, and none had undergone mammogram screening in the past 12 months (Table 1).

**Table 1.** Sociodemographic characteristics of the study participants (N=405).

Variables	Frequency, n (%)
Age groups	
40‐49 years	187 (46.2)
50‐59 years	140 (34.6)
60 years and older	78 (19.2)
Education level	
Primary	123 (30.4)
Secondary	173 (42.7)
University	109 (26.9)
Marital status	
Single	104 (25.7)
Married	213 (52.6)
Divorced	37 (9.1)
Widowed	51 (12.6)
Do you have any health insurance?	
Yes	372 (91.9)
No	33 (8.1)
Mammogram screening within the last 12 months	
Yes	0 (0)
No	405 (100)

The participants’ responses to the open-ended questions were analyzed and categorized into 8 main reasons that may negatively impact screening practices. An example of the main reasons reported among the 405 participants was a lack of knowledge, cited by 72.8% (295). This was followed by fear of results, reported by 66.4% (269), cultural beliefs at 63% (255), and religious reasons, mentioned by 29.4% (119) (Multimedia Appendix 5).

### Knowledge About Breast Cancer

Table 2 shows that the overall knowledge score about BC was relatively low (48.3%), with a mean (SD) score of 5.80 (2.64). Regarding knowledge aspects, approximately out of 405, three-quarters of the sample, 74.3% (301), correctly recognized that BC is the most common cancer among women. In addition, half of the sample, 206 (50.9%), identified excess weight and having a first child after age 30 years as risk factors for BC. In contrast, only 145 (35.8%) correctly identified that most breast tumors are not cancerous.

**Table 2.** Breast cancer knowledge questions with correct answers (N=405).

Item		Correct answer	Correct answer, n (%)
Myths and misconceptions			
A strong blow to the chest can cause breast cancer after a period of time.		False	192 (47.4)
The constant discomfort of a tight bra may cause breast cancer after a period of time.		False	176 (43.5)
Some types of fibrous sacs in the breast (noncancerous tumors) increase the risk of breast cancer.		False	176 (43.5)
Women over the age of 70 rarely have breast cancer.		False	186 (45.9)
Most breast tumors are cancerous.		False	145 (35.8)
Risk factors			
Excess weight may cause breast cancer to some women.		True	195 (48.1%)
A woman who conceives her first child after the age of thirty is more likely to have breast cancer than a woman who conceives her first child before the age of thirty.		True	206 (50.9%)
Women in the United States are more likely to have breast cancer than Asian or African women.		True	167 (41.2)
Breast cancer is more common among women at age 65 than at age 40.		True	188 (46.4)
The most common cancer among women is breast cancer.		True	301 (74.3)
Physical exercises reduce the risk of breast cancer.		True	202 (49.9)
Diet affects the risk of breast cancer.		True	214 (52.8)

### Beliefs About Mammography

Scores for each construct and question within the CHBM scale ranged from 1 to 5. The perceived benefits of mammography had a relatively low mean (SD) score of 2.59 (0.59). Among the perceived benefits, women identified that mammography helps in the early detection of lumps, with a mean (SD0 score of 2.62 (1.08). The perception that mammography decreases the chances of dying from BC had a mean (SD0 score of 2.60 (1.13). Furthermore, the item related to reducing worry about BC was rated the lowest, with a mean (SD) score of 2.45 (1.19) (Table 3).

**Table 3.** Breast cancer knowledge and health beliefs scores based on the Champion Health Belief Model scale.

Items		Mean (SD)
Knowledge		5.80^a^ (2.64)
CHBM^b^ construct		
Perceived benefits		2.59^c^ (0.59)
When I get a recommended mammogram, I feel good about myself.		2.62 (1.10)
When I get a mammogram, I don’t worry as much about breast cancer.		2.45 (1.19)
Having a mammogram of the breast will help me find lumps early.		2.62 (1.08)
Having a mammogram of the breast will decrease my chances of dying from breast cancer.		2.60 (1.13)
Having a mammogram of the breast will decrease my chances of requiring radical or disfiguring surgery if breast cancer occurs.		2.60 (1.14)
Having a mammogram will help me find a lump before it can be felt by myself or a health care professional.		2.62 (1.03)
Perceived barriers^d^		2.48 (0.71)
Having a routine mammogram of the breast would make me worry about breast cancer.		2.52 (0.92)
Having a mammogram of the breast would be embarrassing.		2.38 (1.00)
Having a mammogram of the breast would take too much time.		2.49 (0.96)
Having a mammogram of the breast would be painful.		2.48 (1.02)
Having a mammogram of the breast would cost too much money.		2.54 (1.05)
Health Motivation		2.51 (0.71)
I want to discover health problems early.		2.64 (1.09)
Maintaining good health is extremely important to me.		2.62 (1.12)
I search for new information to improve my health.		2.64 (1.12)
I feel it is important to carry out activities which will improve my health.		2.63 (1.12)
I eat well-balanced meals.		2.61 (1.13)
I exercise at least 3 times a week.		2.31 (0.95)
I have regular health check-ups, even when I’m not sick.		2.11 (1.08)

a Mean score for the knowledge item out of 12.

b CHBM: Champion Health Belief Model

c Mean score for all items of perceived benefits, barriers, and health motivation out of 5.

d Reversed score: a lower score indicates higher perceived barriers.

The perceived barriers to undergoing mammograms were assessed with a mean (SD) score of 2.48 (0.71), indicating that women have various concerns about this procedure. Worries related to BC (item 7) had a mean (SD) score of 2.52 (0.92), while fears of pain had a mean (SD) score of 2.48 (1.02). Item 8, “Having a mammogram of the breast would be embarrassing,” was the highest perceived barrier, with a mean (SD) score of 2.38 (1.00).

Participants reported a low health motivation score regarding mammography, with a mean (SD) of 2.51 (0.71). Among the health motivation items, the desire to detect health problems early (item 12) had the highest mean (SD) score of 2.64 (1.09). In contrast, lower scores were observed for exercising 3 times a week (item 17), with a mean (SD0 of 2.31 (0.95), and for undergoing regular health check-ups even when not sick (item 18), with a mean (SD) of 2.11 (1.08).

### Knowledge and Beliefs Regarding BC and Mammography Across Sociodemographic Characteristics

The statistical analysis, conducted using MANOVA, revealed that age and educational level had a significant influence on knowledge and beliefs, as measured by the CHBM scale (*P*<.05 for all). Moreover, the Bonferroni post hoc tests for age groups and educational levels revealed that women aged 60 years and older with primary or secondary education demonstrated lower levels of knowledge, perceived benefits, and health motivation. In addition, they reported higher perceived barriers compared with women in the 40‐49 years and 50‐59 years age groups who had a university education (*P*<.001). In contrast, marital status and health insurance had no significant effect on the variables related to BC and mammography (*P*>.05 for all) (Table 4).

**Table 4.** Mean differences in knowledge and health beliefs.

Variables		Knowledge			Perceived benefits				Perceived barriers^a^				Health motivation		
		Mean (SD)	*P* value^b^	ηp² ^c^		Mean (SD)	*P* value	ηp²		Mean (SD)	*P* value	ηp²	Mean (SD)	*P* value	ηp²
Age groups (years)			<.001	0.10			<.001	0.122			<.001	0.088		*<*.001	0.080
40‐49		6.43 (2.70)				2.86 (0.97)				2.65 (0.73)			2.68 (0.77)		
50‐59		5.86 (2.44)				2.54 (0.70)				2.48 (0.63)			2.49 (0.62)		
60 years and older		4.17 (2.13)				2.02 (0.68)				2.08 (0.64)			2.14 (0.57)		
Education level			<.001	0.471			*<*.001	0.503			<.001	0.184		<.001	0.371
Primary		3.42 (1.69)				1.77 (0.47)				2.09 (0.66)			1.94 (0.47)		
Secondary		5.98 (1.92)				2.63 (0.60)				2.50 (0.60)			2.56 (0.58)		
University		8.19 (2.15)				3.44 (0.81)				2.89 (0.69)			3.08 (0.64)		
Marital status			.05	0.019			.54	0.005			.41	0.007		.71	0.003
Single		5.75 (2.39)				2.61 (0.92)				2.48 (0.76)			2.45 (0.76)		
Married		6.05 (2.73)				2.62 (0.92)				2.49 (0.70)			2.54 (0.72)		
Divorced		5.70 (2.27)				2.51 (0.76)				2.62 (0.63)			2.51 (0.60)		
Widowed		4.92 (2.84)				2.43 (0.78)				2.36 (0.69)			2.51 (0.66)		
Health insurance			.72	0.000			.94	0.000			.87	0.000		.59	0.000
Yes		5.81 (2.69)				2.58 (0.89)				2.48 (0.71)			2.51 (0.72)		
No		5.64 (1.97)				2.60 (0.85)				2.46 (0.69)			2.53 (0.62)		

a Reversed score: a lower score indicates higher perceived barriers.

b MANOVA test. A *P* value less than .05 was considered significant.

c Partial eta square (ηp²): ηp²<0.01:Negligible, 0.01≤ηp²<0.06:Small, 0.06≤η²<0.14:Medium, ηp²≥0.14: Large.

The results showed that age significantly influenced knowledge (*F*_2402_=22.44; *P*<.001). Participants aged 40‐49 years had a mean (SD) score of 6.43 (2.70), while those aged 50‐59 years had a mean (SD) score of 5.86 (2.44). These groups had significantly higher mean scores than participants aged 60 years and older, who had a mean (SD) score of 4.17 (2.13, *P*<.001). In addition, the educational level also significantly influenced knowledge (*F*_2402_=179.05; *P*<.001). Participants with a university education had mean (SD) scores of 8.19 (2.15), which were higher than those with secondary education, 5.98 (1.92), and primary education, 3.42 (1.69), *P*<.001. In addition, the partial eta squared (ηp²) results for age showed a medium effect size for knowledge (ηp²=0.10), while education level displayed a large effect size for knowledge (ηp²=0.471).

Similarly, perceived benefits were significantly influenced by age (*F*_2402_=27.82; *P*<.001), and had a medium effect size (ηp²=0.122). The mean (SD) score for the participants aged 40‐49 years was 2.86 (0.97), and for those aged 50‐59 years, it was 2.54 (0.70). Both mean scores were higher than those of participants aged 60 years and older, who had a mean (SD) score of 2.02 (0.68), *P*<.001. In addition, the educational level significantly influenced perceived benefits (*F*_2402_=203.11; *P*<.001), and had a medium effect size (ηp²=0.503). Participants with a university education had higher mean (SD) scores of 3.44 (0.81) compared with those with secondary education, 2.63 (0.60), and primary education, 1.77 (0.47), *P*<.001.

Moreover, the perceived barriers were significantly influenced by age (*F*_2402_=19.32; *P*<.001) with a medium effect size (ηp²=0.088). Participants aged 60 years and older displayed significantly higher barrier scores, with a mean (SD) score of 2.08 (0.64), compared with those in the 40‐49 years age group, 2.65 (0.73), and the 50‐59 years age group, 2.48 (0.63), *P*<.001. In addition, the educational level had a significant influence (*F*_2402_=45.24; *P*<.001), and a large effect size (ηp²=0.184). Participants with primary education had higher barrier mean (SD) scores of 2.09 (0.66) compared to those with secondary education, 2.50 (0.60), and university education, 2.89 (0.69), *P*<.001.

Ultimately, health motivation was significantly influenced by age (*F*_2402_=17.53; *P*<.001). The participants’ mean (SD) score for both ages 40‐49 years was 2.68 (0.77), and that for 50‐59 years was 2.49 (0.62), which was higher than those of participants 60 years and older, 2.14 (0.57), *P*<.001. The educational level also had a significant influence (*F*_2402_=118.54; *P*<.001), with women holding a university education 3.08 (0.64) scoring higher than those with secondary education 2.56 (0.58) and primary education 1.94 (0.47), *P*<.001. Furthermore, the partial eta squared results for age revealed a medium effect size for health motivation (ηp²=0.080). The effect size was large for both educational level and health motivation (ηp²=0.371).

Regarding marital status, the effect sizes were small or negligible for all dependent variables: knowledge (ηp²=0.019), perceived benefits (ηp²=0.005), perceived barriers (ηp²=0.007), and health motivation (ηp²=0.003). Finally, there was no effect (ηp²=0.000) on any dependent variables for health insurance status.

## Discussion

### Principal Findings

This cross-sectional study examines the knowledge and beliefs of Jordanian women aged 40 years and older regarding BC and mammography, using the HBM as a theoretical framework. The findings offer valuable insights into the knowledge, perceived benefits, barriers, and health motivations that influence mammogram screening practices among this population.

Participants in this study reported several barriers that adversely affected their participation in screening practices, such as cost, fear of results, pain, cultural beliefs, and religious factors. These findings were consistent with those observed by Abu-Helalah et al in Jordan [14], which identified the main reasons for avoiding screening as fear of results (63.8%), lack of support (59.7%), cost (53.4%), and religious beliefs (51.1%). Another study conducted by AlAbdulKader et al [41] in Saudi Arabia found that the main barriers to receiving BCS include feeling that it is unnecessary (24.2%), believing it to be painful (22.1%), and fearing abnormal results (18.6%). In addition, other regional and global studies support these findings, highlighting fear of pain and fear of diagnosis as common reasons for avoiding BCS [42,43].

Furthermore, the prevalence of cultural beliefs (63%) and religious factors (29.4%) is a significant barrier to mammography screening, as observed in our study and supported by Albadawi et al [44], who found that these beliefs influence BCS behaviors among women in Jordan. Similarly, Taha et al [45] and Salman et al [46] revealed through qualitative interviews that many Jordanian women hold fatalistic views shaped by the religious concept of “Qadaa Wa Qadar” (predestination).

These beliefs lead many to view BC as “from Allah (God)” and thus unavoidable, reducing their perceived benefits of early detection. This religious fatalism, as highlighted in studies by Petro-Nustas et al and Donnelly et al [22,47], serves as a significant barrier to adopting preventive health behaviors. Moreover, Taha et al [45] also discussed this in a study conducted in Jordan, where men viewed BC as an unavoidable act of God, detached from their families. This belief is linked to notions of improper behavior and a readiness to confront the cultural stigma of Eib (shame) [48]. In addition, research by Al-Naggar and Bobryshev [49] found that religious beliefs accounted for 42.5% of screening avoidance among Malaysian Muslim women, closely aligning with our finding of 37.5%. Their study also emphasized that support from religious leaders can boost participation rates, a factor worth exploring in the Jordanian context.

We observed a significant lack of knowledge about BC among the participants in this study, with a low average score of 5.80 out of 12. Furthermore, 72.8% of participants indicated that their lack of knowledge was the primary reason for not undergoing mammogram screenings. This finding aligns with Kawar’s study [50], which emphasized the importance of health care advice in improving awareness and encouraging participation in screenings. In addition, a 2023 national survey conducted among Palestinian women revealed that 53.2% believed the idea of “having a physical trauma” was a myth associated with BC, while 19.4% regarded “wearing a tight bra” as a misconception [51]. In contrast, our study found that only 43.5% of participants understood that not all fibrous sacs increase the risk of BC, and only 35.8% recognized that not all breast tumors are cancerous. These findings suggest a substantial gap in understanding breast abnormalities, which is similar to the health literacy deficiencies commonly observed in low-resource settings, where limited access to accurate health information contributes to poor health literacy [7,52,53].

The findings from the CHBM scale in this study revealed that perceived barriers had the lowest mean (SD) score of 2.48 (0.71) out of 5, followed by health motivation at 2.51 (0.59) out of 5 and perceived benefits at 2.59 (0.71) out of 5. This finding is consistent with previous studies that have identified perceived barriers as the most significant predictor of mammogram screening [27,33,37]. Participants in our study showed a negative perception regarding the benefits of mammography, as evidenced by their lower scores on statements such as: “When I get a mammogram, I do not worry as much about BC” and “Having a mammogram will decrease my chances of dying from BC.” These results were compatible with findings from other studies [22,33], as reported by Al-Naggar and Bobryshev [49], which indicated that Malaysian women with a limited understanding of the benefits of mammography were less likely to participate in regular screenings; only 15% had ever undergone a mammogram. Together, these findings underscore the importance of educational interventions in addressing misconceptions and promoting awareness of the benefits of mammography [54].

In our study, participants reported higher perceived barriers to mammogram screening, such as the belief that “having a mammogram would be embarrassing” or “having a mammogram would be painful.” Previous studies support these findings, as Amin et al [55] found that embarrassment and anxiety about pain significantly deterred Bangladeshi women from mammography screening, especially where modesty is prioritized. In addition, Al-Mousa et al [56] found that Jordanian women often avoid mammography due to feelings of embarrassment and fear of the results, as they believe they do not need a mammogram if they have no family history of BC, which is deeply rooted in cultural beliefs. These studies emphasize the need for culturally sensitive interventions to increase the screening rate.

Moreover, participants in our study had a negative perception of health motivation, with low mean scores for attending regular check-ups, maintaining a balanced diet, and exercising regularly. These findings suggest that targeted health interventions should increase awareness about the importance of routine check-ups, provide accessible and culturally appropriate dietary guidance, and promote exercise through structured programs to foster sustained engagement in preventive health behaviors [22,23,56]. The motivation for health and awareness was significantly associated with screening behavior [27].

Our study’s findings, related to the association between sociodemographic characteristics and knowledge and beliefs regarding BC and mammography, reveal that participants’ age and education have a significant influence on their knowledge and beliefs. Older and less educated women showed lower awareness and more negative perceptions regarding the screening. These results align with previous studies and underscore the significant influence of age and education on shaping women’s perspectives toward mammography screening [23,27,56,57].

Specifically, our study’s findings revealed a significant association between knowledge and beliefs, as measured by the CHBMS, and the ages and educational levels of the participants (*P*<.05). Participants aged 40‐49 years and 50‐59 years scored higher on knowledge, perceived benefits, and health motivation compared with those aged 60 years and older (*P*<.001). This was consistent with previous studies suggesting that younger women have better access to health information, are more exposed to BC awareness campaigns, and benefit from higher literacy rates and greater access to digital platforms [58,59]. Younger women appreciate early detection, which is influenced by targeted screening and health care recommendations. Moreover, older women may underestimate the benefits of mammography, possibly due to rooted cultural beliefs, as found by Mah et al [60] that older women felt less vulnerable to BC than younger women and were less positive about screening. This suggests that motivation declines with age due to health priorities and misconceptions about the effectiveness of screening in older adults [61,62].

Similarly, women with a university education scored higher on knowledge, perceived benefits, and health motivation compared to those with secondary and primary education (*P*<.001). This supports the notion that individuals with higher education are more proactive about their health and preventive care and are more likely to follow medical advice [63]. This suggests that highly educated women are more likely to recognize the value of early detection and preventive care [64]. This suggests a more substantial health motivation for the importance of screening among women with higher levels of education [63,64].

Conversely, the perceived barriers were significantly more negative among participants aged 60 years and older compared with those aged 40‐49 years and 50‐59 years (*P*<.001), aligning with the logistical and psychological challenges faced by older women and their traditional beliefs [65,66]. Furthermore, participants with primary education reported more barriers than those with secondary and university education (*P*<.001), suggesting that lower education is associated with higher perceived obstacles, such as lack of knowledge, fear, embarrassment, financial concerns, and cultural beliefs [67,68].

Neither marital status nor health insurance had a significant impact on knowledge and beliefs (*P*>.05). This finding contrasts with studies suggesting that married women benefit from spousal support for mammography [69,70]. While health insurance facilitates access to screening, its impact may diminish in areas with few financial barriers [71,72].

### Limitations

The study has several limitations. Its cross-sectional design uses convenience sampling, which prevents the determination of causal relationships between beliefs and screening behaviors and limits the generalizability of the results. The reliance on self-reported data may introduce information bias. Recruiting participants from a single hospital through convenience sampling may limit the generalizability of the findings to all Jordanian women aged 40 years and older, as it will not encompass all Jordanian women from different geographical locations, particularly those from rural areas. Furthermore, excluding women who had a mammogram within the last 12 months, as well as those with personal or family histories of BC, may introduce selection bias and limit insights into the influence of hereditary factors on screening behaviors.

### Implications for Practice

Developing effective educational interventions for Jordanian women requires a multifaceted approach that addresses both individual and systemic barriers. These interventions must account for varying levels of awareness, health beliefs, and the influence of religious factors, such as fatalism, which affect screening behaviors. Involving religious and community leaders in program content can boost motivation and reduce participation barriers.

Educational initiatives should include different community activities, such as workshops, home visits, and outreach sessions with community leaders, religious figures, and BC survivors. This approach fosters trust and delivers culturally appropriate information. Expanding mobile mammography units and organizing workplace screening campaigns are crucial, especially for underserved rural populations.

Public health officials and health care providers need ongoing training in culturally competent communication and patient counseling to ensure sensitive and supportive care. To improve accessibility, financial barriers can be addressed by offering free or subsidized mammograms for low-income women and advocating for broader insurance coverage.

Delivery methods should use digital health tools, such as mobile apps and SMS text messaging reminders, which can enhance personalized education and appointment alerts. In addition, providing culturally appropriate support services, including flexible scheduling and female-only environments, is important for addressing concerns about modesty and privacy.

These interventions should be integrated within a robust policy framework that updates national screening guidelines and incorporates educational efforts into a national registry and monitoring system. By tackling awareness levels, health beliefs, and religious perspectives, Jordan can implement effective interventions to improve BCS and outcomes for women nationwide. Ultimately, ongoing research to monitor screening trends is crucial for evaluating and enhancing health initiatives, providing valuable insights to inform future policies and programs.

### Conclusion

This study contributes to the growing body of research on BCS in Jordan and similar contexts. It highlights significant barriers to mammogram screening among Jordanian women aged 40 years and older, including a lack of knowledge, cultural beliefs, and psychological and financial challenges. The study emphasizes the importance of establishing a solid foundation for culturally appropriate and sensitive interventions that are tailored to the local context and targeted to improve health awareness, accessibility, and policy reforms. Health care providers and policy makers can adopt an evidence-based approach to enhance early detection, reduce BC mortality rates, and promote a healthier future. Future research should focus on assessing the long-term effects of these initiatives.

## Supplementary material

10.2196/75384 Multimedia Appendix 1 Mammography Screening Questionnaire.

10.2196/75384 Multimedia Appendix 2 Permission to view and modify the Health Belief Model.

10.2196/75384 Multimedia Appendix 3 Ethical Approval.

10.2196/75384 Multimedia Appendix 4 Consent form.

10.2196/75384 Multimedia Appendix 5 Table: Reasons for not getting Mammogram screening within the last 12 months

10.2196/75384 Checklist 1 Strengthening the Reporting of Observational studies in Epidemiology checklist.

## Abbreviations

ACR: American College of Radiology
ASR: age-standardized rate
BC: breast cancer
BCS: breast cancer screening
CHBM: Champions Health Belief Model
CHBMS: Champions Health Belief Model Scale
HBM: Health Belief Model
IRB: Institutional Review Board
JBCP: Jordan Breast Cancer Program
LMIC: lower-middle-income country
MANOVA: multivariate analysis of variance
STROBE: STrengthening the Reporting of OBservational studies in Epidemiology

## References

[1] Hastings KG, Boothroyd DB, Kapphahn K (2018). Socioeconomic differences in the epidemiologic transition from heart disease to cancer as the leading cause of death in the United States, 2003 to 2015: an observational study. Ann Intern Med.

[2] Population factsheets. International Agency for Research on Cancer.

[3] Bray F, Laversanne M, Sung H (2024). Global cancer statistics 2022: GLOBOCAN estimates of incidence and mortality worldwide for 36 cancers in 185 countries. CA Cancer J Clin.

[4] (2024). Breast cancer. World health organization.

[5] Francies FZ, Hull R, Khanyile R, Dlamini Z (2020). Breast cancer in low-middle income countries: abnormality in splicing and lack of targeted treatment options. Am J Cancer Res.

[6] (2025). Country classifications by income. World Bank.

[7] Abdel-Razeq H, Mansour A (2024). Challenges and opportunities in breast cancer care in low-resourced countries, Jordan as an example. Cancers (Basel).

[8] Cancer J. Registry (2022). Jordan Cancer Registry annual report. Jordan Ministry of Health.

[9] Promoting Breast Cancer Awareness and Early Detection. Jordan breast cancer program.

[10] Lambertini M, Santoro L, Del Mastro L (2016). Reproductive behaviors and risk of developing breast cancer according to tumor subtype: a systematic review and meta-analysis of epidemiological studies. Cancer Treat Rev.

[11] Abdel-Razeq H, Mansour A, Jaddan D (2020). Breast cancer care in Jordan. JCO Glob Oncol.

[12] Monticciolo DL, Newell MS, Moy L, Lee CS, Destounis SV (2023). Breast cancer screening for women at higher-than-average risk: updated recommendations from the ACR. J Am Coll Radiol.

[13] (2022). Cancer facts & figures for african american/black people 2022-2024. American Cancer Society.

[14] Abu-Helalah MA, Alshraideh HA, Al-Serhan AA, Kawaleet M, Nesheiwat AI (2015). Knowledge, barriers and attitudes towards breast cancer mammography screening in jordan. Asian Pac J Cancer Prev.

[15] (2018). Cancer incidence in jordan. Jordan Ministry of Health.

[16] Obeidat RF, Lally RM (2023). Determinants of breast cancer screening mammography uptake: Results from Jordan population and family health survey. Indian J Cancer.

[17] Bener A, El Ayoubi HR, Moore MA, Basha B, Joseph S, Chouchane L (2009). Do we need to maximise the breast cancer screening awareness? Experience with an endogamous society with high fertility. Asian Pac J Cancer Prev.

[18] Al-Zalabani AH, Alharbi KD, Fallatah NI, Alqabshawi RI, Al-Zalabani AA, Alghamdi SM (2018). Breast cancer knowledge and screening practice and barriers among women in Madinah, Saudi Arabia. J Cancer Educ.

[19] Quintin C, Chatignoux E, Plaine J, Hamers FF, Rogel A (2022). Coverage rate of opportunistic and organised breast cancer screening in France: Department-level estimation. Cancer Epidemiol.

[20] Melvin CL, Jefferson MS, Rice LJ, Cartmell KB, Halbert CH (2016). Predictors of participation in mammography screening among non-Hispanic Black, non-Hispanic White, and Hispanic women. Front Public Health.

[21] Gupta A, Shridhar K, Dhillon PK (2015). A review of breast cancer awareness among women in India: Cancer literate or awareness deficit?. Eur J Cancer.

[22] Taha H, Halabi Y, Berggren V (2010). Educational intervention to improve breast health knowledge among women in Jordan. Asian Pac J Cancer Prev.

[23] Alkhasawneh IM (2007). Knowledge and practice of breast cancer screening among Jordanian nurses. Oncol Nurs Forum.

[24] Areej Othman RN, Ahram M, Obeidat RF, Obeidat N, Rasoul Al-Tarawneh M (1097). Barriers for mammography among non-adherent women in Jordan: a national survey. Life Sci.

[25] Ferreira CS, Rodrigues J, Moreira S, Ribeiro F, Longatto-Filho A (2021). Breast cancer screening adherence rates and barriers of implementation in ethnic, cultural and religious minorities: A systematic review. Mol Clin Oncol.

[26] Petro-Nustas W (2001). Young Jordanian women’s health beliefs about mammography. J Community Health Nurs.

[27] Othman AK, Kiviniemi MT, Wu YW, Lally RM (2012). Influence of demographic factors, knowledge, and beliefs on Jordanian women’s intention to undergo mammography screening. J Nurs Scholarsh.

[28] Rosenstock IM (1974). The health belief model and preventive health behavior. Health Educ Monogr.

[29] Alyafei A, Easton-Carr R (2025). StatPearls.

[30] Becker MH, Maiman LA (1980). Strategies for enhancing patient compliance. J Community Health.

[31] Champion VL (1984). Instrument development for health belief model constructs. ANS Adv Nurs Sci.

[32] Champion VL (1993). Instrument refinement for breast cancer screening behaviors. Nurs Res.

[33] Champion VL (1999). Revised susceptibility, benefits, and barriers scale for mammography screening. Res Nurs Health.

[34] Champion VL, Springston J (1999). Mammography adherence and beliefs in a sample of low-income African American women. Int J Behav Med.

[35] Mikhail BI, Petro-Nustas WI (2001). Transcultural adaptation of champion’s health belief model scales. J Nurs Scholarsh.

[36] von Elm E, Altman DG, Egger M, Pocock SJ, Gøtzsche PC, Vandenbroucke JP (2008). The strengthening the reporting of observational studies in epidemiology (STROBE) statement: guidelines for reporting observational studies. J Clin Epidemiol.

[37] Saei Ghare Naz M, Simbar M, Rashidi Fakari F, Ghasemi V (2018). Effects of model-based interventions on breast cancer screening behavior of women: a systematic review. Asian Pac J Cancer Prev.

[38] Matlabi H, Asgari Z, Morsali Asl S, Mousavi S, Rezakhani Moghaddam H (2021). The effectiveness of health belief model initiative in breast cancer screening behaviors among women health volunteers. Soc Work Public Health.

[39] Sample size calculator. Raosoft, inc.

[40] (2021). SPSS statistics for windows version 280. IBM corporation.

[41] AlAbdulKader A, Gari D, Al Yousif G (2023). Perceived Barriers and Facilitators to Breast Cancer Screening Among Women in Saudi Arabia. Breast Cancer (Dove Med Press).

[42] Ayaad O, Al Ajmi AA, Al Baimani K (2025). Breast Cancer Awareness, Screening Practices, Barriers, and Educational Interventions in Middle Eastern Countries: Challenges and Successes. Asian Pac J Cancer Biol.

[43] Kawar LN (2013). Barriers to breast cancer screening participation among Jordanian and Palestinian American women. Eur J Oncol Nurs.

[44] Albadawi RS, Alsharawneh A, Othman EH (2025). Determinants and barriers to women’s participation in breast cancer screening activities in Jordan: an in-depth study. BMC Public Health.

[45] Taha H, Al-Qutob R, Nyström L, Wahlström R, Berggren V (2012). “Voices of fear and safety” women’s ambivalence towards breast cancer and breast health: a qualitative study from Jordan. BMC Womens Health.

[46] Salman K, Zoucha R, Nawafleh H (2018). Understanding Jordanian women’s values and beliefs related to breast cancer: a focused ethnography. J Transcult Nurs.

[47] Donnelly TT, Al Khater AH, Al-Bader SB (2013). Beliefs and attitudes about breast cancer and screening practices among Arab women living in Qatar: a cross-sectional study. BMC Womens Health.

[48] Taha H, Al-Qutob R, Nyström L, Wahlström R, Berggren V (2013). “Would a man smell a rose then throw it away?” Jordanian men’s perspectives on women’s breast cancer and breast health. BMC Womens Health.

[49] Al-Naggar RA, Bobryshev YV (2012). Practice and barriers of mammography among Malaysian women in the general population. Asian Pac J Cancer Prev.

[50] Kawar LN (2012). Knowledge about breast cancer and negative influences affecting breast cancer screening among women in Jordan. IJHSS.

[51] Elshami M, Ismail IO, Alser M (2023). Common myths and misconceptions about breast cancer causation among Palestinian women: a national cross-sectional study. BMC Public Health.

[52] Atrooz F, Aljararwah SM, Acquati C, Khabour OF, Salim S (2023). Breast cancer beliefs and screening practices among Syrian refugee women and Jordanian women. Int J Environ Res Public Health.

[53] Othman A, Ahram M, Al-Tarawneh MR, Shahrouri M (2015). Knowledge, attitudes and practices of breast cancer screening among women in Jordan. Health Care Women Int.

[54] Rezaeian M, Sharifirad G, Mostafavi F, Moodi M, Abbasi MH (2014). The effects of breast cancer educational intervention on knowledge and health beliefs of women 40 years and older, Isfahan, Iran. J Educ Health Promot.

[55] Amin MN, Uddin MG, Uddin MN (2020). A hospital based survey to evaluate knowledge, awareness and perceived barriers regarding breast cancer screening among females in Bangladesh. Heliyon.

[56] Al-Mousa DS, Spuur K, Attar R, Kleib I, Alakhras M (2024). Knowledge, attitudes, and practices related to breast cancer screening among female Jordanian university employees: A cross-sectional study. Radiography (Lond).

[57] Damiani G, Basso D, Acampora A (2015). The impact of level of education on adherence to breast and cervical cancer screening: Evidence from a systematic review and meta-analysis. Prev Med.

[58] Eun Y, Lee EE, Kim MJ, Fogg L (2009). Breast cancer screening beliefs among older Korean American women. J Gerontol Nurs.

[59] Friedman LC, Neff NE, Webb JA, Latham CK (1998). Age-related differences in mammography use and in breast cancer knowledge, attitudes, and behaviors. J Cancer Educ.

[60] Mah Z, Bryant H (1992). Age as a factor in breast cancer knowledge, attitudes and screening behaviour. CMAJ.

[61] Dolan NC, Lee AM, McDermott MM (1997). Age-related differences in breast carcinoma knowledge, beliefs, and perceived risk among women visiting an academic general medicine practice. Cancer.

[62] Safizade H, Amirzadeh N, Mangolian Shahrbabaki P (2020). Motivational factors for breast cancer screening behaviors in Iranian women: a qualitative study. Asian Pac J Cancer Prev.

[63] Gürdal SÖ, Saraçoğlu GV, Oran EŞ, Yankol Y, Soybir GR (2012). The effects of educational level on breast cancer awareness: a cross-sectional study in Turkey. Asian Pac J Cancer Prev.

[64] Rasu RS, Rianon NJ, Shahidullah SM, Faisel AJ, Selwyn BJ (2011). Effect of educational level on knowledge and use of breast cancer screening practices in Bangladeshi women. Health Care Women Int.

[65] Abdel-Aziz SB, Amin TT, Al-Gadeeb MB (2018). Perceived barriers to breast cancer screening among Saudi women at primary care setting. J Prev Med Hyg.

[66] Joho AA, Mdoe MB, Masoi TJ, Yahaya JJ (2024). Perceived barriers and factors influencing uptake of breast cancer screening among women: a population-based cross-sectional study. Sci Rep.

[67] Abugu LI, Nwagu EN, Okeke AI, Odo AN (2023). Knowledge of breast cancer, willingness and barriers to mammography screening among rural women in Enugu State, Nigeria. Afr Health Sci.

[68] Olsson SE, Shah S, Haase E, Butler E, Amado I, Pagidas K (2025). Perceptions and barriers to screening mammography and clinical breast examination: a survey study of underserved populations in North Texas. Public Health Chall.

[69] Hanske J, Meyer CP, Sammon JD (2016). The influence of marital status on the use of breast, cervical, and colorectal cancer screening. Prev Med.

[70] Hinyard L, Wirth LS, Clancy JM, Schwartz T (2017). The effect of marital status on breast cancer-related outcomes in women under 65: A SEER database analysis. Breast.

[71] Bitler MP, Carpenter CS (2016). Health insurance mandates, mammography, and breast cancer diagnoses. Am Econ J Econ Policy.

[72] Levy AR, Bruen BK, Ku L (2012). Health care reform and women’s insurance coverage for breast and cervical cancer screening. Prev Chronic Dis.

