# Supplementary material for: Knowledge and Beliefs Toward Mammography Screening Among Jordanian Women: Cross-Sectional Study
Source: JMIR Public Health Surveill. 2025 Aug 21;11:e75384. doi: 10.2196/75384 (PMC12370264; doi:10.2196/75384)
Supplement: Multimedia Appendix 1 [file publichealth-v11-e75384-s001.docx]

| **Filled by Data Collector** | | | |
| --- | --- | --- | --- |
|  | Questionnaire  Number |  | Participant Phone Number |
|  | Date |  | Data-Collector Code |

**Mammography Screening Questionnaire**

**First Part: Sample Characteristics**

1. **Age (years):……………**
2. **Educational level:**

### □Primary Education

□Secondary Education

□University Education

1. **Marital Status:**

### □Single

### □Married

### □Divorced

### □Widow

1. **Do you have any health insurance?**

### □Yes

### □No

1. **A mammogram is an X-ray of the breast for early detection of breast cancer. Have you done this test within the last 12 months?**

### □Yes

### □No

1. **If the previous answer is No, what are the most important reasons for not getting this test?**

### ……………………………………………

### ……………………………………………

### ……………………………………………

### ……………………………………………

**Second Part: Knowledge About Breast Cancer:**

1. A strong blow to the chest can cause breast cancer after a period of time.

A- True B- False C-1 Don't Know

1. The constant discomfort of a tight bra may cause breast cancer after a period of time.

A- True B- False C-1 Don't Know

1. Excess weight may cause breast cancer to some women.

A- True B- False C-1 Don't Know

1. A woman who conceives her first child after the age of thirty is more likely to have breast cancer than a woman who conceives her first child before the age of thirty.

A- True B- False C-1 Don't Know

1. Some types of fibrous sacs in the breast (noncancerous tumors) increase the risk of breast cancer.

A- True B- False C-1 Don't Know

1. Women in the United States are more likely to have breast cancer than Asian or African women.

A- True B- False C-1 Don't Know

1. Breast cancer is more common among women at age 65 than at age 40.

A- True B- False C- I Don't Know

1. The most common cancer among women is breast cancer.

A- True B- False C-1 Don't Know

1. Women over the age of 70 rarely have breast cancer.

A- True B- False C-1 Don't Know

1. Most breast tumors are cancerous.

A- True B- False C- I Don't Know

1. Physical exercises reduce the risk of breast cancer.

A- True B- False C-1 Don't Know

1. Diet affects the risk of breast cancer.

A- True B- False C-1 Don't Know

**Third Part: Health Beliefs:**

***BENEFITS-MAMMOGRAM***

1. When I get a recommended mammogram, I feel good about myself.

1. Strongly Disagree 2. Disagree 3. Neutral 4. Agree 5. Strongly-Agree

1. When I get a mammogram, I don't worry as much about breast cancer.

1. Strongly Disagree 2. Disagree 3. Neutral 4. Agree 5. Strongly-Agree

1. Having a mammogram of the breast will help me find lumps early.

1. Strongly Disagree 2. Disagree 3. Neutral 4. Agree 5. Strongly-Agree

1. Having a mammogram of the breast will decrease my chances of dying from breast cancer.

1. Strongly Disagree 2. Disagree 3. Neutral 4. Agree 5. Strongly-Agree

1. Having a mammogram of the breast will decrease my chances of requiring radical or disfiguring surgery if breast cancer occurs.

1. Strongly Disagree 2. Disagree 3. Neutral 4. Agree 5. Strongly-Agree

1. Having a mammogram will help me find a lump before it can be felt by myself or a health profession.

1. Strongly Disagree 2. Disagree 3. Neutral 4. Agree 5. Strongly-Agree

***BARRIERS-MAMMOGRAM***

1. Having a routine mammogram of the breast would make me worry about breast cancer.

1. Strongly Disagree 2. Disagree 3. Neutral 4. Agree 5. Strongly-Agree

1. Having a mammogram of the breast would be embarrassing.

1. Strongly Disagree 2. Disagree 3. Neutral 4. Agree 5. Strongly-Agree

1. Having a mammogram of the breast would take too much time.

1. Strongly Disagree 2. Disagree 3. Neutral 4. Agree 5. Strongly-Agree

1. Having a mammogram of the breast would be painful.

1. Strongly Disagree 2. Disagree 3. Neutral 4. Agree 5. Strongly-Agree

1. Having a mammogram of the breast would cost too much money.

1. Strongly Disagree 2. Disagree 3. Neutral 4. Agree 5. Strongly-Agree

***HEALTH MOTIVATION***

1. I want to discover health problems early.

1. Strongly Disagree 2. Disagree 3. Neutral 4. Agree 5. Strongly-Agree

1. Maintaining good health is extremely important to me.

1. Strongly Disagree 2. Disagree 3. Neutral 4. Agree 5. Strongly-Agree

1. I search for new information to improve my health.

1. Strongly Disagree 2. Disagree 3. Neutral 4. Agree 5. Strongly-Agree

1. I feel it is important to carry out activities which will improve my health.

1. Strongly Disagree 2. Disagree 3. Neutral 4. Agree 5. Strongly-Agree

1. I eat well-balanced meals.

1. Strongly Disagree 2. Disagree 3. Neutral 4. Agree 5. Strongly-Agree

1. I exercise at least 3 times a week.

1. Strongly Disagree 2. Disagree 3. Neutral 4. Agree 5. Strongly-Agree

1. I have regular health check-ups even with I am not sick.

1. Strongly Disagree 2. Disagree 3. Neutral 4. Agree 5. Strongly-Agree
