# Supplementary material for: Knowledge and Beliefs Toward Mammography Screening Among Jordanian Women: Cross-Sectional Study
Source: JMIR Public Health Surveill. 2025 Aug 21;11:e75384. doi: 10.2196/75384 (PMC12370264; doi:10.2196/75384)
Supplement: Multimedia Appendix 2 [file publichealth-v11-e75384-s002.pdf]

INDIANA UNIVERSITY
SCHOOL OF NURSING

June 24, 2024

Ahmad Abu Abed, PhD Student
University of Granada

Dear Ahmad Abu Abed,

Thank you for your interest in my work. You have permission to view and modify the Health Belief Model for your use as long as you cite my work and send me an abstract of your completed projects.

Sincerely,

Victoria Champion, PhD, RN, FAAN
Mary Margaret Walther Distinguished Professor
Edward W. and Sarah Stam Cullipher Endowed Chair
Assistant Director of Cancer Control and Population Science
Indiana University Simon Cancer Center

600 Barnhill Indianapolis, IN 46202-5107 (317) 274-4187 fax (317) 278-2021
