# Supplementary figures and images for: Knowledge and Beliefs Toward Mammography Screening Among Jordanian Women: Cross-Sectional Study
Source: JMIR Public Health Surveill. 2025 Aug 21;11:e75384. doi: 10.2196/75384 (PMC12370264; doi:10.2196/75384)

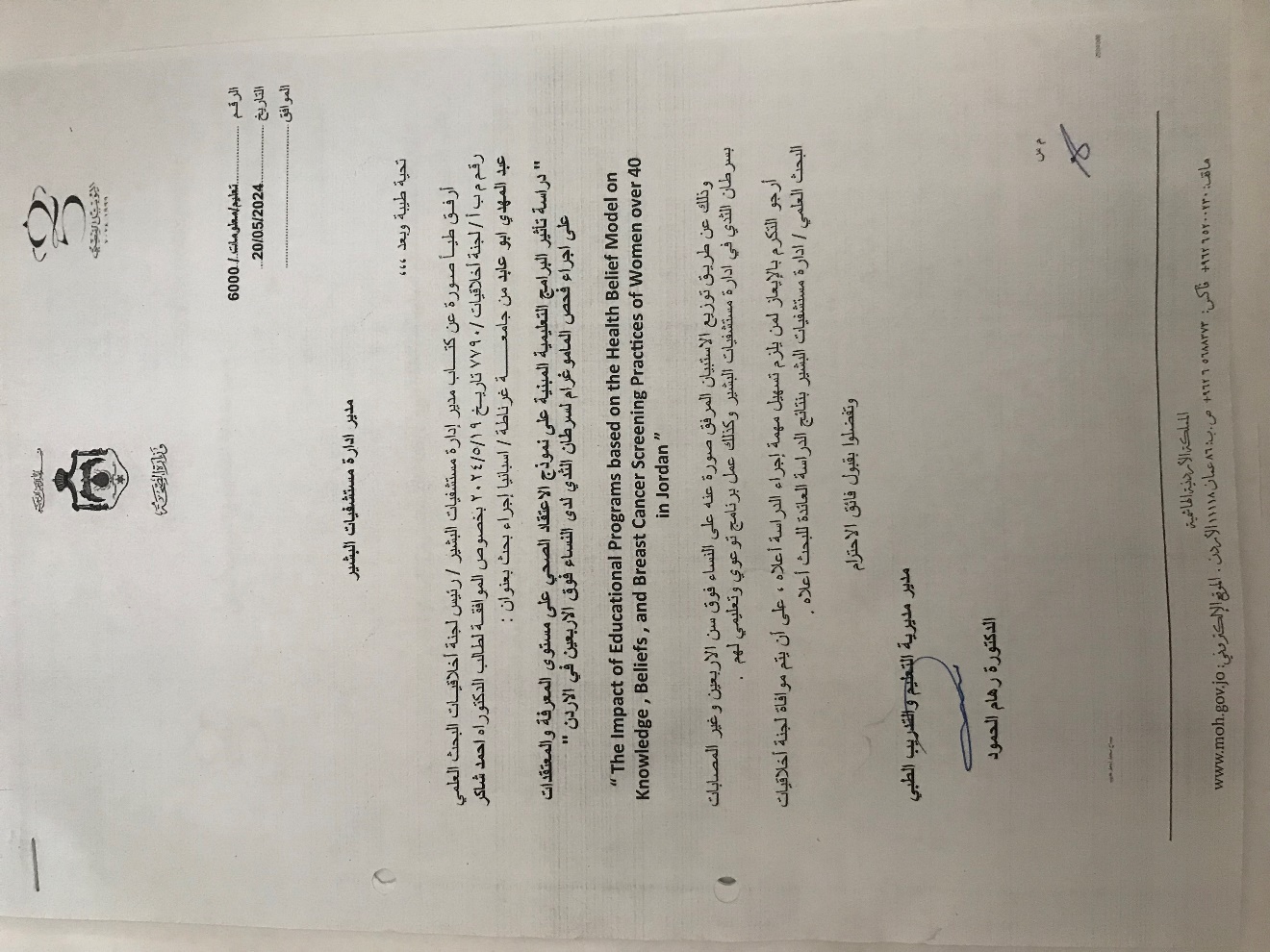


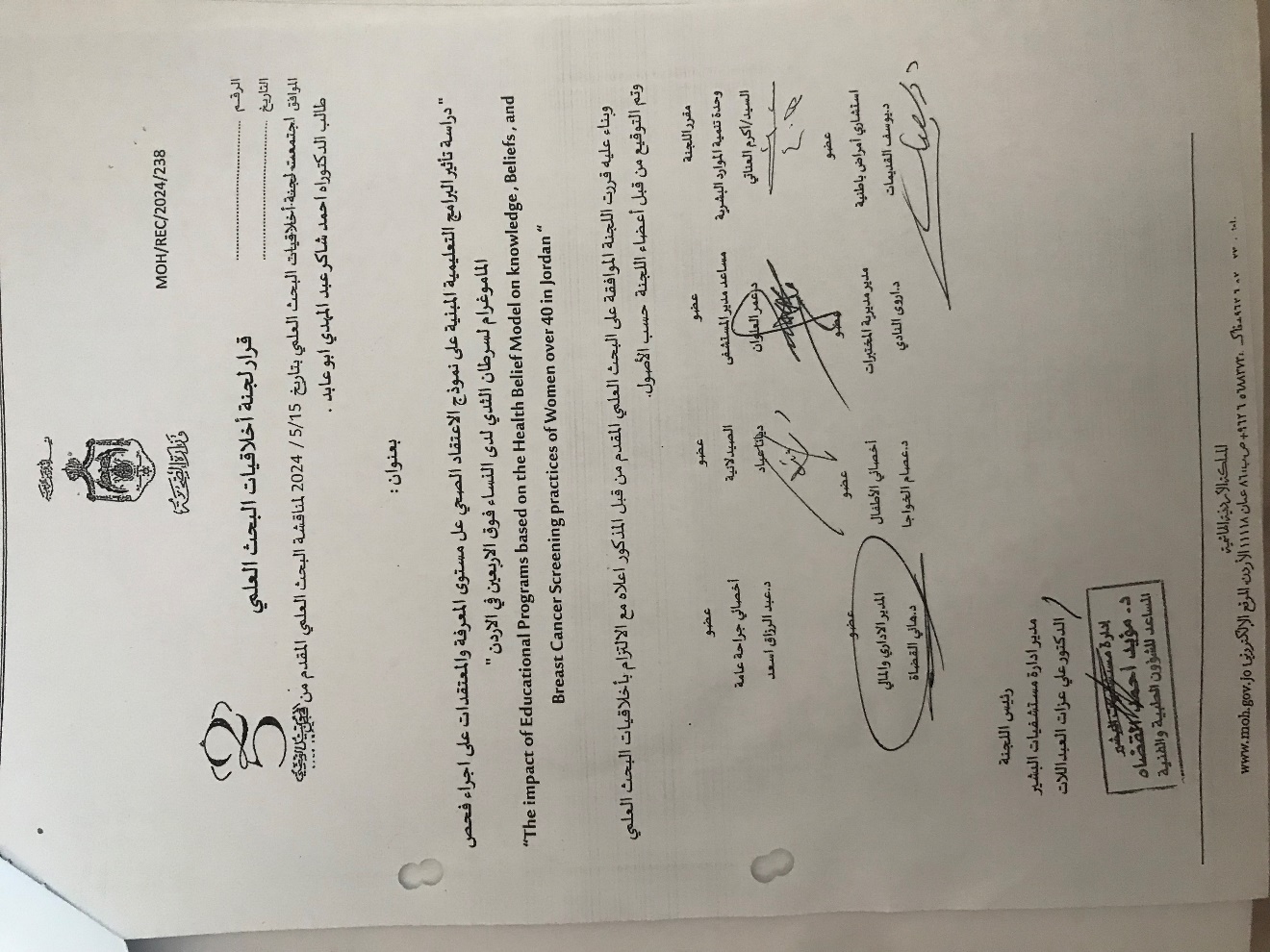


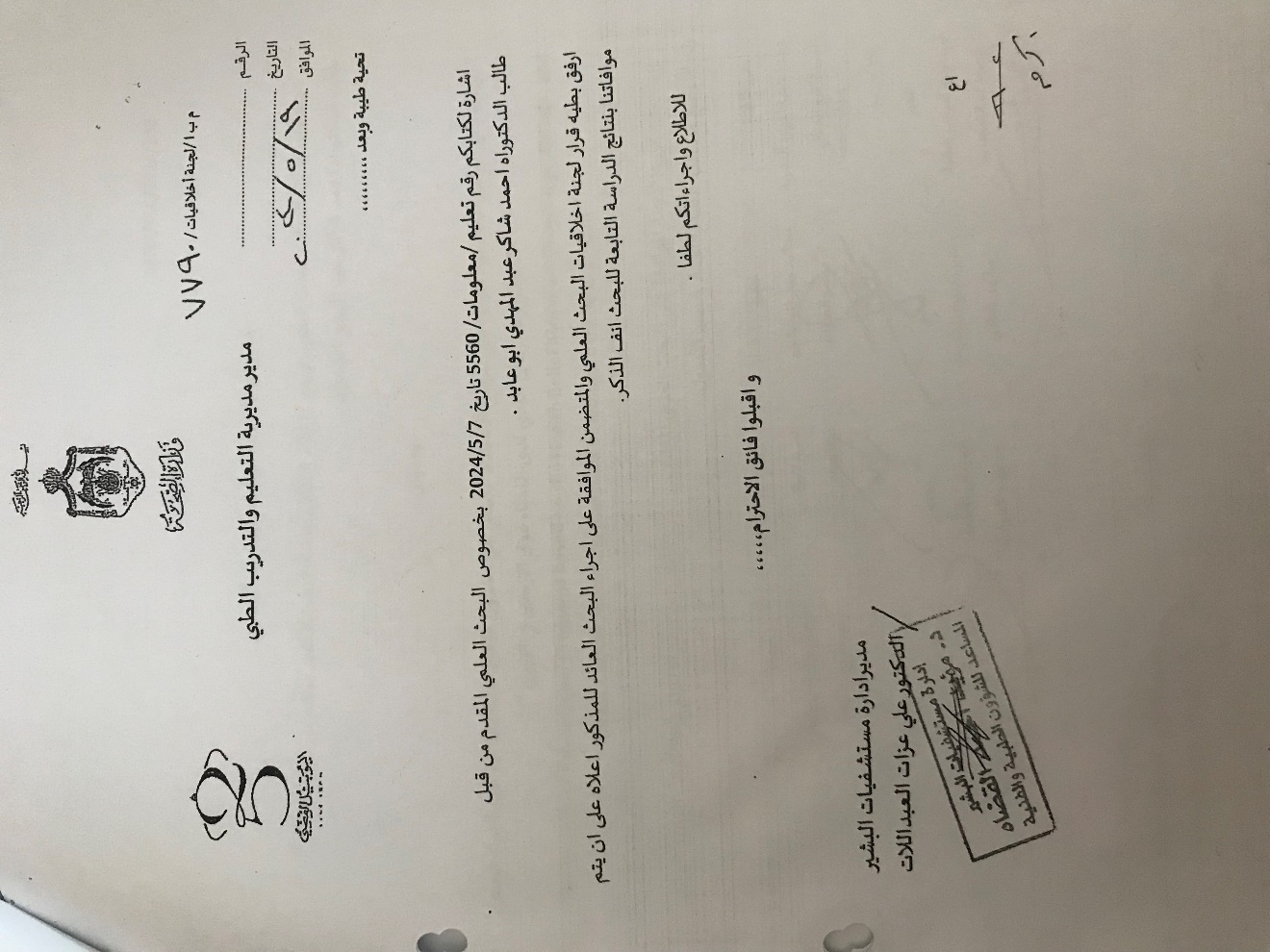

Supplement: Multimedia Appendix 3 [file publichealth-v11-e75384-s003.docx]
