# Supplementary material for: Knowledge and Beliefs Toward Mammography Screening Among Jordanian Women: Cross-Sectional Study
Source: JMIR Public Health Surveill. 2025 Aug 21;11:e75384. doi: 10.2196/75384 (PMC12370264; doi:10.2196/75384)
Supplement: Multimedia Appendix 4 [file publichealth-v11-e75384-s004.docx]

**Consent Form**

**Dear Participant**

You are invited to participate in a research study conducted by Mr. Ahmad Abuabed from the University of Granada, Spain. This study aims to evaluate the level of knowledge and beliefs related to breast cancer and mammogram screening among women in Jordan. The results will be used to develop an awareness and educational program about breast cancer.

The questionnaire will take approximately 15 minutes to complete. Your responses will be kept confidential, and your personal information will not be disclosed to anyone outside the research team. Participation in this study is voluntary, and you have the right to withdraw at any time without any penalty. Your decision to participate will not affect your medical care or any other services you receive.

If you have any questions or concerns regarding this study, please do not hesitate to contact Mr. Ahmad Abuabed via email at ahmadabuabed820@gmail.com or by phone at - 00962 79543 7376.

By signing below, you indicate that you have read and understood the information provided above. You voluntarily agree to participate in this study.

**Participant’s Signature**: ___________________________

**Date**: ___________________________

**Thank you for your participation.**
