# Supplementary material for: Knowledge and Beliefs Toward Mammography Screening Among Jordanian Women: Cross-Sectional Study
Source: JMIR Public Health Surveill. 2025 Aug 21;11:e75384. doi: 10.2196/75384 (PMC12370264; doi:10.2196/75384)
Supplement: Multimedia Appendix 5 [file publichealth-v11-e75384-s005.docx]

**Multimedia Appendix 5**. Reasons for not getting Mammogram screening within the last 12 months

| Items | Frequency distribution and percentage | | | | | | |
| --- | --- | --- | --- | --- | --- | --- | --- |
| Reasons for not getting Mammogram screening within the last 12 months | Reason | Frequency  Reason1 | Frequency  Reason2 | Frequency  Reason3 | Frequency  Reason4 | Total | Percentage^a^ (%) |
|  | **Knowledge** | 102 | 73 | 68 | 52 | 295 | 72.8 |
|  | **Fear-Result** | 87 | 68 | 58 | 56 | 269 | 66.4 |
|  | **Culture** | 64 | 60 | 64 | 67 | 255 | 63 |
|  | **Fear-Pain** | 55 | 52 | 53 | 70 | 230 | 56.8 |
|  | **Time** | 33 | 39 | 40 | 47 | 159 | 39.2 |
|  | **H.C. Access** | 25 | 38 | 43 | 45 | 151 | 37.3 |
|  | **Cost** | 21 | 38 | 43 | 40 | 142 | 35.8 |
|  | **Religion** | 18 | 37 | 36 | 28 | 119 | 29.4 |
|  | Total | 405 | 405 | 405 | 405 |  | |

^a^ The percentage for each reason was calculated by dividing the total frequency of that reason, reported by each participant across all four reasons, by the total sample size of 405 participants.
